# Topological Alterations of the Brain Functional Network in Type 2 Diabetes Mellitus Patients With and Without Mild Cognitive Impairment

**DOI:** 10.3389/fnagi.2022.834319

**Published:** 2022-04-19

**Authors:** Baiwan Zhou, Xia Wang, Qifang Yang, Faqi Wu, Lin Tang, Jian Wang, Chuanming Li

**Affiliations:** ^1^Department of Radiology, The Second Affiliated Hospital of Chongqing Medical University, Chongqing, China; ^2^Department of Radiology, Southwest Hospital, Third Military Medical University, Chongqing, China; ^3^Department of Medical Service, Yanzhuang Central Hospital of Gangcheng District, Jinan, China

**Keywords:** type 2 diabetes mellitus, mild cognitive impairment, resting-state functional magnetic resonance imaging, graph theory, functional connectome

## Abstract

The aim of this study was to explore the topological alterations of the brain functional network in type 2 diabetes mellitus (T2DM) patients with and without mild cognitive impairment (MCI) using resting-state functional magnetic resonance imaging (rs-fMRI) and graph theory approaches. In total, 27 T2DM patients with MCI, 27 T2DM patients without MCI, and 27 healthy controls (HCs) underwent rs-fMRI scanning. The whole-brain functional network was constructed by thresholding the Pearson’s correlation matrices of 90 brain regions. The topological organization of the constructed networks was analyzed by using graph theory approaches. The global and nodal properties of the participants in the three groups were compared by using one-way ANOVA as well as *post hoc* Tukey’s *t*-tests. The relationships between the altered topological properties and clinical features or scores of neuropsychological tests were analyzed in T2DM patients with MCI. At the global level, the global and local efficiency of the patients in the T2DM with MCI group were significantly higher than that of participants in the HCs group, and the length of the characteristic path was significantly lower than that of the participants in the HCs group (*p* < 0.05). No significant difference was found among the other groups. At the nodal level, when compared with T2DM patients without MCI, T2DM patients with MCI showed significantly increased nodal centrality in four brain regions, which were mainly located in the orbitofrontal lobe and anterior cingulate gyrus (ACG) (*p* < 0.05). No significant difference was found between the T2DM patients without MCI and HCs. Moreover, nodal degree related coefficient (*r* = −0381, *p* = 0.050) and nodal efficiency (*r* = −0.405, *P* = 0.036) of the ACG showed a significant closed correlation with the scores of the digit span backward test in the T2DM patients with MCI. Our results suggested that the increased nodal properties in brain regions of the orbitofrontal lobe and ACG were biomarkers of cognitive impairment in T2DM patients and could be used for its early diagnosis. The global topological alterations may be related to the combination of MCI and T2DM, rather than any of them.

## Introduction

Type 2 diabetes mellitus (T2DM) is a metabolic disease characterized by long-term hyperglycemia, insulin resistance, and a relative lack of insulin, which increases the risk for cognitive impairment (McCrimmon et al., 2012). Approximately 10.8–17.5% of T2DM patients show various cognitive impairments (Hoyer, 1998; Bruce et al., 2003; Moran et al., 2013), such as mild cognitive impairment (MCI) and dementia. Compared to dementia, MCI is a reversible alteration under certain circumstances, where 29–55% of patients with MCI could return to normal (Koepsell and Monsell, 2012). Early diagnosis and timely treatment are helpful to slow or cure the progression of MCI to dementia (Sanford, 2017). It is very important to study the mechanism of cognitive impairment in T2DM patients and to identify new biomarkers to promote its early diagnosis.

As a non-invasive neuroimaging technique, magnetic resonance imaging (MRI) has been widely used in the study of the pathogenesis of neurological and mental diseases. Previous MRI studies have suggested that compared to healthy individuals, patients with T2DM show alterations in gray matter volume (Moran et al., 2015), white matter (WM) volume (Reijmer et al., 2013b), cortical thickness (Brundel et al., 2010), and spontaneous brain activity (Cui et al., 2014) in specific brain regions. Simultaneously, gray matter atrophy (Groeneveld et al., 2018), WM atrophy (Li et al., 2020a), and decreased cortical thickness (Li et al., 2018) have also been reported in T2DM patients with MCI. However, these studies mainly focused on regional measures. When completing a task, the human brain needs the interaction and coordination of various functional regions. Studying the topological structure formed by the interaction of various functional regions could help further elucidate the mechanism of functional impairment in the brain. For example, Huang et al. (2019) found that T2DM patients displayed significant alterations in the node properties of certain brain regions, and an anterior-posterior disconnection phenotype and altered topological configuration of the default mode network (DMN) were also observed in patients with T2DM (Chen et al., 2016). Regarding functional networks in T2DM patients with MCI, an increased clustering coefficient and decreased normalized characteristic path length have been reported (Xu et al., 2019). Qi et al. also found stronger DMN functional connectivity in the left precuneus and weaker functional connectivity in the left calcarine (Qi et al., 2017). For structural networks, Li et al. (2020b) reported lower nodal efficiency and fewer connections in multiple brain regions of T2DM patients with MCI. However, most of the literature assumes that the signal change is directly determined by T2DM, while the role of MCI is ignored. It is particularly important to study whether the topological alterations are actually related to T2DM, MCI, or their joint effect, and to investigate new biomarkers of cognitive impairment.

In this study, we used resting-state functional MRI (rs-fMRI) and graph theory analysis to explore the differences in functional network topology among T2DM patients with MCI, T2DM patients without MCI, and healthy controls (HCs) to deepen our understanding of the pathogenesis of MCI in T2DM and help its early diagnosis.

## Materials and Methods

### Participants

Patients diagnosed with T2DM were recruited from our hospital between March 2015 and June 2016. T2DM was diagnosed according to the criteria proposed by the Alberti and Zimmet (1998). MCI was diagnosed by the criteria presented by Petersen (Portet et al., 2006), which included (1) complaints of hypomnesis; (2) a Mini-Mental State Examination (MMSE) score greater than 24 and a Montreal Cognitive Assessment (MoCA) score less than 26; (3) no significant alteration in the daily living score; and (4) no dementia. The exclusion criteria included (1) other neurological or psychiatric illnesses, (2) contraindications to MRI, and (3) obvious microvascular disease. The neuropsychological assessments included the MMSE, MoCA, Auditory Verbal Learning Test (AVLT), Complex Figure Test (CFT), Verbal Fluency Test (VFT), Trail Making Test (TMT), Digit Symbol-coding Test (DSCT), Digit Span Backward Test (DSBT), and Digit Span Forward Test (DSFT). The healthy controls (HCs), who had no history of nervous system disease, vascular risk factors, cognitive complaints, or psychiatric illness, were recruited through advertisements. In total, 54 T2DM patients, including 27 T2DM patients with MCI and 27 T2DM patients without MCI, and 27 successfully matched HCs were included in our study.

### Data Acquisition and Preprocessing

Resting-state functional MRI data were obtained from a 3T MRI scanner (MAGNETOM Trio Tim System, Siemens, Erlangen, Germany) with a 12-channel head coil. When the participants were scanned, they were asked to keep their eyes closed and stay awake. Each examination lasted for 300 s and contained 144 image volumes. We tried to minimize the influences of head motion by using foam pads. The parameters included repetition time (TR) ms/echo time (TE) ms, 2000/30; flip angle, 90°; thickness = 3 mm, gap = 1 mm, layer number = 36; matrix = 64 × 64; and voxel size, 3.5 mm^3^ × 3.5 mm^3^ × 3 mm^3^. All the subjects were also required to undergo conventional brain T1-weighted and fluid-attenuated inversion recovery (FLAIR) imaging to exclude organic diseases and WM hyperintense lesions. The T1-weighted images were obtained using the following parameters: TR/TE = 200/2.78 ms, flip angle = 70°, matrix = 384 × 384, thickness = 4.0 mm, 25 slices, and voxel size = 0.7 mm^3^ × 0.6 mm^3^ × 5 mm^3^. The FLAIR sequence was scanned using the following parameters: TR/TE/inversion time (TI) = 9000/93/2500 ms, flip angle = 130°, matrix = 256 × 256, thickness = 4.0 mm, 25 slices, and voxel size = 0.9 mm^3^ × 0.9 mm^3^ × 4 mm^3^.

Data preprocessing was performed using Gretna version 2.0 software.^1^ First, the original 10 time points were deleted to minimize the impact of signal instability at the beginning of the MRI scan, and the correction was carried out for the acquisition delay between slices. Friston 24-parameter correction and “head motion scrubbing” were used to ensure that the effects of head motion did not contribute to the results that we obtained. For every participant, volumes with framewise displacement (FD) beyond 0.2 mm or derivative variance greater than 0.5% of the BOLD signal were identified and excluded, and these time points were then interpolated with nearest neighbor spline interpolation. The individual mean FD value was also treated as a within-subjects nuisance factor in group-level analyses. After these corrections, images were normalized with a voxel size of 3 mm^3^ × 3 mm^3^ × 3 mm^3^ and smoothed by using a 4 mm full-width at half-maximum (FWHM) Gaussian kernel. Finally, a filter (bandpass: 0.01–0.1 Hz) was used to minimize the influences of low-frequency drift and high-frequency noise.

### Construction of the Functional Network and Topological Analysis

This step was performed by using Gretna version 2.0 software. First, we separated the whole brain into 90 cortical or subcortical regions based on the anatomical automatic labeling (AAL) atlas (Fenchel et al., 2008). Each region was regarded as a node of the whole-brain functional network. The edge was defined by the correlation coefficient between the mean time series of two arbitrary nodes, which was calculated by using Pearson’s correlation analysis. Finally, we obtained a 90 × 90 undirected and weighted correlation matrix for each subject.

A series of sparsity thresholds (0.1–0.34) was then set for each correlation matrix. All topological properties were calculated by using the area under the curve (AUC) beyond each sparsity threshold, which could minimize the influence of the threshold diversity. Seven global properties and two nodal properties were computed. The global properties mainly contained (1) small-world parameters (Watts and Strogatz, 1998), including clustering coefficient (Cp), characteristic path length (Lp), normalized clustering coefficient (γ), normalized characteristic path length (λ), and small-worldness (σ), and (2) network efficiency parameters (Latora and Marchiori, 2001), including local efficiency (Eloc) and global efficiency (Eglob). The nodal properties mainly included the nodal degree (Rubinov and Sporns, 2010) and nodal efficiency (Achard and Bullmore, 2007).

### Connection Analysis

The functional connections between brain regions with significant differences in nodal properties among the three groups were identified by using a network-based statistical (NBS) method (*p* < 0.05, T threshold > 1.62). Then, we can identify a subnetwork composed of nodes with altered nodal properties and edges with altered connections.

### Correlation Analysis

The associations between topological alterations and clinical features [including body mass index (BMI), fasting glucose, glycosylated hemoglobin (HbA1c), and duration of diabetes] or the scores of neuropsychological tests (including AVLT, CFT, VFT, TMT, DSCT, DSBT, DSFT, MoCA, and MMSE) of the T2DM patients with MCI were analyzed by using the Pearson correlation analysis method (*p* < 0.05).

### Statistical Analysis

One-way ANOVA was used to analyze the differences in each topological property among the three groups (T2DM patients with MCI, T2DM patients without MCI, and HCs) with a false discovery rate (FDR)-corrected alpha threshold of 0.05. When there was a significant difference among the three groups, a *post hoc* Tukey’s *t*-test with a threshold of 0.05 was used for pairwise comparison.

## Results

### Participants

Participants in the three groups showed no significant difference in age, sex, education years, BMI, disease duration, AVLT-5 min delayed recall score, AVLT-20 min delayed recall score, CFT-copy score, VFT score, TMT-B score, or MMSE score. Significant differences were detected in AVLT-immediate recall, AVLT-recognition, CFT-immediate recall, CFT-20 min delayed recall, TMT-A, DSCT, DSBT, DSFT, and MoCA scores among the participants in the three groups. Compared with HCs, the patients in the T2DM without MCI group had higher DSCT, DSBT, and DSFT scores, and compared with the patients in the T2DM without MCI group, the patients in the T2DM with MCI group had higher TMT-A scores and lower AVLT-immediate recall, AVLT-recognition, CFT-immediate recall, CFT-20 min delayed recall, and MoCA scores. The demographic and clinical features of the participants in the three groups are shown in Table 1, and the scores on the neuropsychological tests are presented in Table 2.

**TABLE 1:** Demographic and clinical data*^a^*.

Variables	T2DM with MCI	T2DM without MCI	HCs	*P*
Sample size	27	27	27	–
Age (years)*^b^*	54.9 ± 6.0	55.4 ± 5.6	53.6 ± 6.6	0.551*
Sex (M/F)	12/15	16/11	13/14	0.285‡
Education (years)	10.6 ± 3.0	11.6 ± 2.8	11.6 ± 2.8	0.370*
BMI	25.5 ± 3.3	24.5 ± 2.7	23.7 ± 2.6	0.074*
Fasting glucose	8.9 ± 2.2	8.2 ± 1.6	5.5 ± 0.6	< 0.001*
HbA1c	9.2 ± 2.1	8.9 ± 1.7	5.5 ± 0.4	< 0.001*
Disease duration (years)	7 ± 5.5	8 ± 5.9	–	0.442^†^

^a^Data are presented as the mean ± standard deviation. ^b^Age was defined at the time of MRI scanning. *P-values for comparisons among three groups obtained by using one-way ANOVA. ^‡^P-values obtained by using the χ^2^ test among the three groups. ^†^P-values were obtained by using a two-sample t-test. T2DM with MCI, type 2 diabetes mellitus patients with cognitive impairment; T2DM without MCI, type 2 diabetes mellitus patients without cognitive impairment; HCs, healthy controls; BMI, body mass index; HbA1c, glycosylated hemoglobin.

**TABLE 2:** Neuropsychological test outcomes.

Variables	T2DM-MCI	T2DM-N	HCs	P	*Post hoc* test		
					T2DM-MCI vs. HCs	T2DM-MCI vs. T2DM-N	T2DM-N vs. HCs
AVLT-immediate recall	19.0 ± 4.1	23.3 ± 3.5	22.5 ± 5.0	**0.001**	**0.012**	**0.002**	0.789
AVLT-5 min delayed recall	6.9 ± 2.2	8.0 ± 1.5	8.0 ± 1.8	0.064	**0.090**	0.121	0.989
AVLT-20 min delayed recall	6.6 ± 2.5	7.6 ± 1.6	7.7 ± 2.0	0.092	0.105	0.198	0.945
AVLT-recognition	21.2 ± 2.1	22.0 ± 1.5	22.9 ± 1.4	**0.002**	0.065	**0.049**	0.991
CFT-copy	31.7 ± 3.9	32.3 ± 4.2	33.1 ± 1.9	0.303	0.274	0.799	0.636
CFT-immediate recall	18.5 ± 6.6	22.1 ± 7.0	23.9 ± 8.6	**0.032**	**0.027**	0.190	0.660
CFT-20 min delayed recall	18.1 ± 6.3	21.4 ± 7.1	23.5 ± 7.7	**0.027**	**0.021**	0.231	0.532
VFT	41.0 ± 7.2	44.8 ± 8.3	44.1 ± 5.6	0.128	0.253	0.140	0.945
TMT-A	62.4 ± 21.9	52.1 ± 17.9	49.1 ± 17.0	**0.036**	**0.037**	0.136	0.834
TMT-B	78.5 ± 26.6	68.1 ± 24.5	61.6 ± 23.4	0.053	**0.043**	0.290	0.621
DSCT	36.1 ± 8.5	40.4 ± 10.6	45.5 ± 11.8	**0.006**	**0.004**	0.289	0.190
DSBT	4.7 ± 0.9	5.0 ± 0.9	5.5 ± 1.2	**0.008**	**0.006**	0.109	0.528
DSFT	9.0 ± 1.1	8.9 ± 0.8	9.6 ± 1.4	**0.048**	0.172	0.818	**0.047**
MoCA	23.0 ± 1.8	27.0 ± 0.8	27.7 ± 1.2	**<0.001**	**<0.001**	**<0.001**	0.119
MMSE	27.8 ± 1.3	28.3 ± 1.0	28.4 ± 1.1	0.085	0.095	0.191	0.934

*Data are presented as the mean ± SDs. p < 0.05. Comparisons of neuropsychological tests among the threegroups (p < 0.05, shown in bold) and post hoc pairwise comparisons (p < 0.05, shown in bold) were performed using Tukey’s t-test. T2DM-MCI, type 2 diabetes mellitus patients with cognitive impairment; T2DM-N, type 2 diabetes mellitus patients without cognitive impairment; HCs, healthy controls; AVLT, Auditory Verbal Learning Test; CFT, Complex Figure Test; VFT, Verbal Fluency Test; TMT, Trail Making Test; DSCT, Digit Symbol-coding test; DSBT, Digit Span Backward Test; DSFT, Digit Span Forward Test; MoCA, Montreal Cognitive Assessment; MMSE, Mini-Mental State Examination.*

### Global Topological Alterations of the Functional Network

**FIGURE 1:**
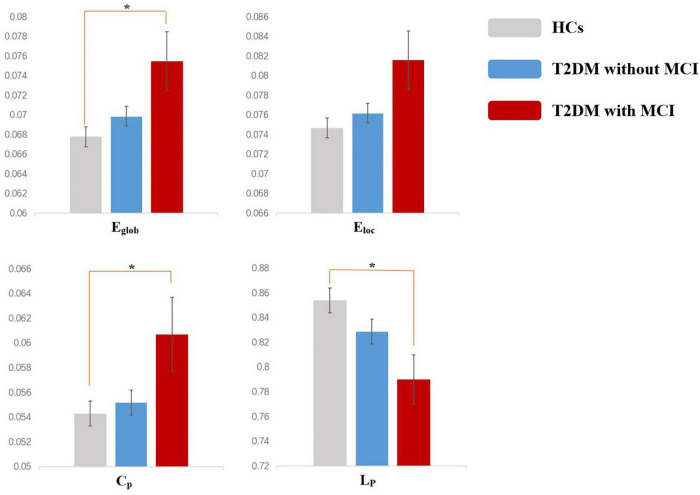
Altered global topologic parameters in the functional network of type 2 diabetes mellitus (T2DM) patients with and without mild cognitive impairment (MCI) (*p* < 0.05). Asterisks indicate significant differences between the two groups in *post hoc* analysis (*p* < 0.05). Error bars represent standard error.

For global properties, significant differences were found in Eglob (*p* = 0.031), Eloc (*p* = 0.048), Cp (*p* = 0.031), and Lp (*p* = 0.041) among the three groups. *Post hoc* analysis suggested that the Eglob (*p* = 0.029) and Cp (*p* = 0.039) in patients in the T2DM with MCI group increased significantly, while Lp (*p* = 0.033) decreased significantly compared with that of the HCs. No significant difference in global properties was detected between the T2DM patients without MCI and the HCs or between the T2DM patients with MCI and the T2DM patients without MCI (Figure 1).

### Nodal Topological Alterations of the Functional Network

The nodes showing significant group differences in both nodal degree and nodal efficiency were considered nodes with altered nodal centrality. The results of one-way ANOVA showed that there were eight brain regions with altered nodal centrality (*p* < 0.05). Compared with the T2DM patients without MCI, T2DM patients with MCI showed increased nodal centrality in four regions, mainly located in the orbitofrontal lobe and anterior cingulate gyrus (ACG) (*p* < 0.05). Compared with the HCs, the T2DM patients with MCI showed increased nodal centrality in eight regions. No significant difference was found between the T2DM patients without MCI and the HCs (Table 3).

**TABLE 3:** Regional measures showing significant differences among participants in the T2DM-MCI, T2DM-N, and HCs groups.

		*Post hoc* tests		
Measurements	*p*(F)	T2DM-MCI vs. HCs	T2DM-MCI vs. T2DM-N	T2DM-N vs. HCs
**Nodal degree**				
Hippocampus_L	**0.003**	**0.002**	0.050	0.519
Superior frontal gyrus, orbital part _R	**0.003**	**0.007**	**0.009**	0.996
Hippocampus_R	**0.003**	**0.002**	0.252	0.132
Inferior temporal gyrus _L	**0.007**	**0.007**	0.061	0.696
Superior occipital gyrus _R	**0.008**	**0.019**	**0.020**	1.000
Inferior frontal gyrus, orbital part_L	**0.010**	**0.011**	0.060	0.785
Parahippocampal gyrus_L	**0.010**	**0.009**	0.097	0.616
Anterior cingulate gyrus _R	**0.012**	**0.024**	**0.030**	0.996
Superior frontal gyrus, orbital part _L	**0.015**	**0.039**	**0.026**	0.986
Supplementary motor area _L	**0.024**	0.065	**0.033**	0.960
Caudate nucleus _R	**0.045**	**0.035**	0.366	0.470
Middle temporal gyrus _R	**0.046**	**0.044**	0.177	0.797
Paracentral lobule _L	**0.046**	**0.035**	0.401	0.438
**Nodal efficiency**				
Hippocampus_L	**0.002**	**0.002**	0.055	0.434
Superior frontal gyrus, orbital part _R	**0.003**	**0.005**	**0.012**	0.962
Hippocampus_R	**0.004**	**0.002**	0.193	0.201
Anterior cingulate gyrus _R	**0.005**	**0.010**	**0.019**	0.971
Inferior temporal gyrus _L	**0.006**	**0.006**	**0.041**	0.762
Inferior frontal gyrus, orbital part_L	**0.008**	**0.008**	0.063	0.078
Superior frontal gyrus, orbital part _L	**0.009**	**0.029**	**0.015**	0.967
Superior occipital gyrus _R	**0.012**	**0.023**	**0.032**	0.991
Paracentral lobule_R	**0.037**	**0.026**	0.420	0.355
Temporal Pole, middle temporal gyrus_L	**0.045**	**0.039**	0.237	0.994
Olfactory cortex _L	**0.046**	0.051	0.136	0.897

*Comparisons of regional measures among participants in the three groups (p < 0.05, false-positive rate corrected, shown in bold) and post hoc pairwise comparisons (p < 0.05, shown in bold) were performed using Tukey’s t-test. Please refer to Supplementary Figure 1 for more details. T2DM-MCI, type 2 diabetes mellitus patients with mild cognitive impairment; T2DM-N, type 2 diabetes mellitus patients without mild cognitive impairment; HCs, healthy controls; L, left; R, right.*

### Alterations in Functional Connections

**FIGURE 2:**
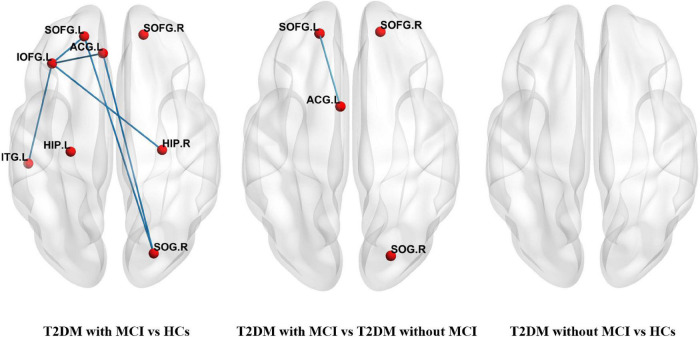
Networks with significant alterations among T2DM patients with MCI, T2DM patients without MCI, and healthy controls (HCs). Each node denoted a brain region with increased nodal centralities, and each edge denoted increased connectivity between these regions. (SOFG, superior frontal gyrus, orbital part; IOFG, inferior frontal gyrus, orbital part; ACG, anterior cingulate gyrus; HIP, hippocampus; SOG, superior occipital gyrus; ITG, inferior temporal gyrus, L-left, R-right).

Compared to the T2DM patients without MCI, the T2DM patients with MCI showed a significantly altered network composed of four nodes and one edge, which were mainly located in the orbitofrontal lobe and ACG. Compared with the HCs, the T2DM patients with MCI had a significantly altered network composed of eight nodes and six edges. No significant difference was found between the T2DM patients without MCI and HCs. The details are presented in Figure 2.

### Correlation Analysis Between Increased Topological Properties and Clinical Features or Scores on Neuropsychological Tests

**FIGURE 3:**
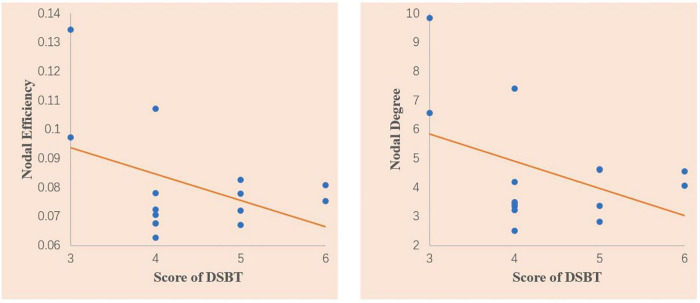
Scatter plots of the correlations between the nodal degree and the Digit Span Backward test (DSBT) score and nodal efficiency of the anterior cingulate gyrus and DSBT score in the T2DM patients with MCI. The DSBT score was significantly negatively correlated with the nodal degree and nodal efficiency of the ACG. Linear model fitting is also shown over the scatterplot (orange line).

In the correlation analysis, we found that nodal degree (*r* = −0381, *p* = 0.050) and nodal efficiency (*r* = −0.405, *p* = 0.036) of the ACG showed a significant closed negative correlation with the DSBT scores of the T2DM patients with MCI (Figure 3). No other correlation was found between the altered topological properties and the clinical features or between the topological properties and neuropsychological scores.

## Discussion

In graph theory, the human brain is defined as a “small world” network with two main organizational principles: segregation, measured by the parameters Cp and Eloc, and integration, measured by the parameters Lp and Eglob (Latora and Marchiori, 2001). Topological segregation refers to the ability of densely interconnected brain regions to perform specialized processing procedures, while integration refers to the efficiency of the ability to combine distributed information in the network or global information communication (Watts and Strogatz, 1998). Both of these aspects are considered to be associated with cognitive function (Vlooswijk et al., 2011). In previous studies, Reijmer et al. (2013a) found a decreased clustering coefficient and increased shortest path length in the WM networks of T2DM patients. Zhang et al. (2014) reported decreased local efficiency and global efficiency in patients with T2DM. In our study, by dividing T2DM patients into subgroups of T2DM patients with MCI and T2DM patients without MCI, we found that differences in global properties were detected only between T2DM patients with MCI and HCs but not between T2DM patients without MCI and HCs or between T2DM patients with MCI and T2DM patients without MCI. These results suggested that the global topological alterations may be related to the combination of MCI and T2DM, rather than any of them. Global topological alterations are not a feature of T2DM or MCI and cannot be used for early diagnosis. A possible explanation for why these alterations cannot be used in early diagnoses is that global topological measures may not be sensitive enough to detect minor changes at the early disease stage. For example, normal topological measures have also been reported in patients with subjective cognitive decline (Chen et al., 2021), which is considered as the prodromal stage of Alzheimer’s disease (AD), while significant abnormalities occur in the AD stages.

To our knowledge, this is the first study to investigate MCI-related topological alterations in T2DM patients. We found alterations in a subnetwork mainly composed of the orbitofrontal lobe and ACG in T2DM patients with MCI compared with T2DM patients without MCI, while no significant difference was detected between the participants in the T2DM without MCI and HC groups. Our results suggested that the functional alterations of the orbitofrontal lobe and ACG in T2DM patients with MCI may be related to MCI rather than T2DM. Increased nodal degree and nodal efficiency in these regions could be used as biomarkers of MCI to promote diagnosis. The orbitofrontal lobe was considered to be associated with the function of decision-making and emotion regulation (Kable and Glimcher, 2007). The ACG has been considered to be related to executive function and attentional control (Lovstad et al., 2012). Thus, dysfunction of the orbitofrontal lobe and ACG will lead to various cognitive impairments (Kennerley et al., 2006; Burke et al., 2008).

In this study, increased nodal properties in T2DM patients with MCI were found, and these properties showed a significant correlation with DSBT scores. The DSBT is widely used as a measurement of working memory by acoustically or optically presented stimuli (Hilbert et al., 2015). It mainly reflects the subjects’ attention control function. The increased nodal properties may represent functional compensation. Previously, increased rs-fMRI signals have also been observed in various studies. For example, more connectivity recruitment between the amygdala and cerebellar regions and between the amygdala and temporal regions has been detected in multiple sclerosis patients (Koubiyr et al., 2021); in addition, increased nodal centralities in the nodes of the cingulo-opercular network, occipital network, and the ventral lateral prefrontal cortex were detected in Parkinson’s disease patients with MCI (Hou et al., 2020).

The present study has several limitations that should be considered. The main limitation is the relatively small sample size. Second, our study is a cross-sectional study. Although we found topological alterations in brain functional networks and their relationship with MCI and T2DM, the dynamics of the relationship are still unclear. Future studies should use a larger sample to conduct a longitudinal study to explore the dynamic relationship among T2DM, MCI, and topology changes.

## Conclusion

In conclusion, in this study, we found that the increased nodal degree and nodal efficiency in the orbitofrontal lobe and ACG were biomarkers of cognitive impairment in T2DM patients. The global topological alterations may be related to the combination of MCI and T2DM, rather than any of them. Our research could improve the present understanding of the mechanism of cognitive impairment in T2DM patients and facilitate its early diagnosis.

## Data Availability Statement

The raw data supporting the conclusions of this article will be available from the corresponding author by request.

## Ethics Statement

The studies involving human participants were reviewed and approved by the Institutional Review Board of Southwest Hospital, Third Military Medical University. The patients/participants provided their written informed consent to participate in this study.

## Author Contributions

BZ and XW designed the study and wrote the manuscript. QY, FW, and JW collected the data. LT processed the data. CL revised the manuscript. All authors reviewed the manuscript and approved the final version to be published.

## Conflict of Interest

The authors declare that the research was conducted in the absence of any commercial or financial relationships that could be construed as a potential conflict of interest.

## Publisher’s Note

All claims expressed in this article are solely those of the authors and do not necessarily represent those of their affiliated organizations, or those of the publisher, the editors and the reviewers. Any product that may be evaluated in this article, or claim that may be made by its manufacturer, is not guaranteed or endorsed by the publisher.

## Footnotes

1
http://www.nitrc.org/projects/gretna/
