# Supplementary material for: Topological Alterations of the Brain Functional Network in Type 2 Diabetes Mellitus Patients With and Without Mild Cognitive Impairment
Source: Front Aging Neurosci. 2022 Apr 19;14:834319. doi: 10.3389/fnagi.2022.834319 (PMC9063631; doi:10.3389/fnagi.2022.834319)

Figure 1. Node degree and Nodal efficency in the functional network of the T2DM with and without MCI patients, as well as HCs.

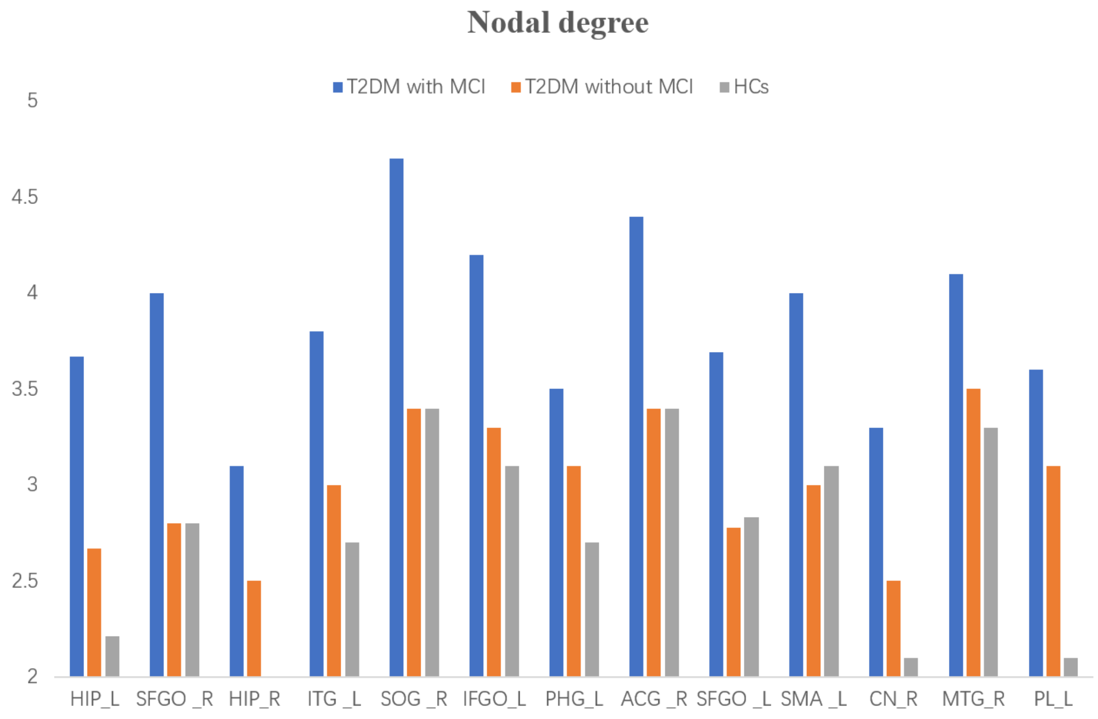

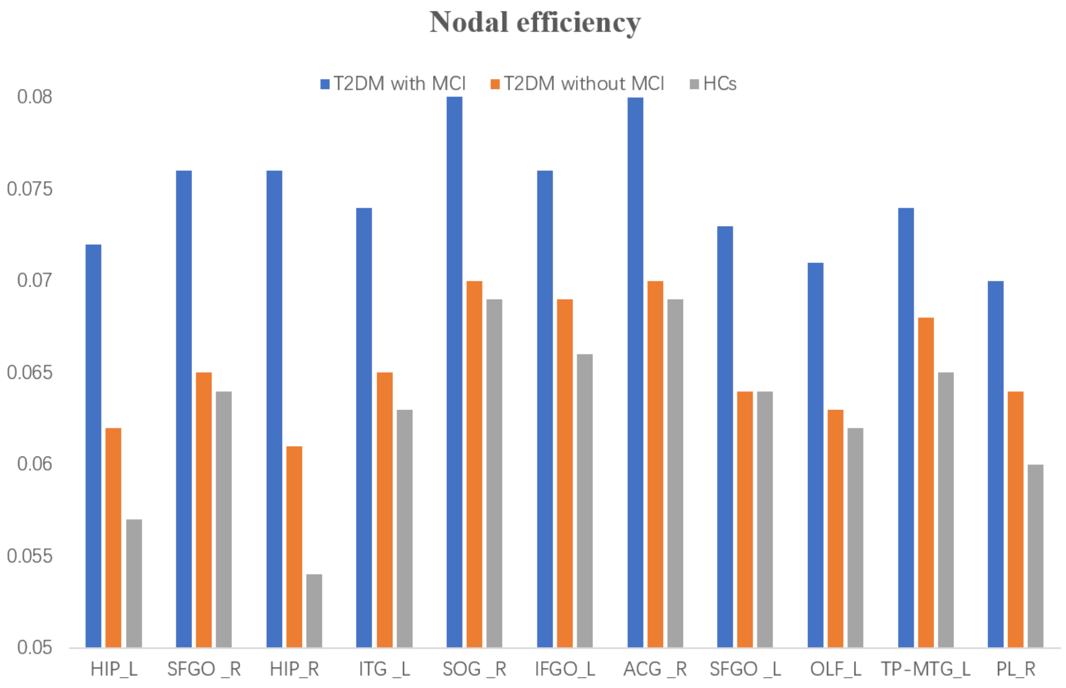

Supplement: Supplementary file 1 [file Data_Sheet_1.PDF]
